# Radiofrequency Ablation for Colorectal Cancer Liver Metastases: Outcomes and Prognostic Factors Associated with Survival

**DOI:** 10.5152/tjg.2023.22088

**Published:** 2023-06-01

**Authors:** Okan Akhan, Seray Akçalar, Emre Ünal, Yavuz Metin, Türkmen Çiftçi, Devrim Akıncı

**Affiliations:** 1 Department of Radiology, Hacettepe University Faculty of Medicine, Ankara, Turkey; 2 Department of Radiology, Kent Health Group, İzmir, Turkey; 3 Department of Radiology, Ankara University Faculty of Medicine, Ankara, Turkey

**Keywords:** Colorectal metastasis, liver, radiofrequency ablation, locoregional therapy

## Abstract

**Background:**

To determine the long-term outcomes of radiofrequency ablation with respect to overall survival, disease-free survival, and complications in patients with colorectal cancer liver metastases. Additionally, we sought to examine whether various patient- and treatment-related characteristics were associated with prognosis.

**Methods:**

Fifty-nine patients with colorectal cancer liver metastases who had undergone percutaneous radiofrequency ablation treatment were included in this study. A total of 138 lesions were treated with radiofrequency ablation in the first and second sessions. Tumor diameters ranged from 10 to 60 mm (mean, 2.45 mm). Treatment efficacy, complications, and overall survival and disease-free survival were analyzed.

**Results:**

The primary success rate of radiofrequency ablation was 94.4%. At the end of the first month, the residual disease was detected in 12 lesions, 10 of which underwent secondary radiofrequency ablation treatment, resulting in a cumulative secondary success rate of 98.4%. The 1-, 3-, and 5-year overall survival rates in 59 patients with colorectal cancer liver metastases were 94.9%, 52.5%, and 40.6%, respectively. The median survival was 42 months in patients with metastasis size of ≤3 cm, while it was 25 months in patients with metastasis size of >3 cm (*P* = .001). The 1-, 3-, and 5-year disease-free survival rates were 44%, 10.2%, and 6.7%, respectively. Metastatic tumor status (solitary or multiple) was a significant prognostic factor in determining overall survival and disease-free survival; furthermore, extrahepatic recurrence during follow-up was a prognostic factor affecting overall survival. Minor complications developed in four radiofrequency ablation procedures (6.7%).

**Conclusion:**

Radiofrequency ablation remains a safe and effective treatment option improving survival in select cases of colorectal cancer liver metastases.

**Main Points**

Image-guided radiofrequency ablation has been long used safely for treatment of metastatic liver lesions of colorectal cancer cases.
RFA is a safe and effective method that prolongs both disease free survival and overall expected survival of suitable patients with metastatic colorectal cancer.
Among available palliative treatment options, RFA is a reliable option with low mortality and morbidity rates without causing any impairment of liver function.
Lesion diameter, tumor status (solitary and multiple) presence of extrahepatic recurrence at initial RFA were found main prognostic factors for overall survival. Tumor status (solitary and multiple) at initial RFA was found main prognostic factors for disease free survival in our study.

## INTRODUCTION

Colorectal cancer (CRC) is the third most common malignancy worldwide and the second most common cause of cancer-related death in industrialized countries.^1^ Colorectal cancer liver metastases (CRCLM) are detected at the time of diagnosis in approximately 25% of patients, while 20% of patients will develop liver metastases during the course of the disease.^2,3^ Total surgical resection of liver metastasis is the only and gold standard treatment for CRCLM^3,4^ and has been shown to yield a 5-year overall survival (OS) rate varying between 27% and 58%.^2,5^ and a median survival time of 28-46 months.^6^ Hence, parenchyma-sparing surgery with a negative surgical margin is the gold standard treatment to increase survival among patients with operable CRC who have liver metastasis.^7^ Unfortunately, 75%-90% of these patients are unsuitable for surgical resection due to various reasons, including the extent and site of the lesion, the presence of vascular invasion, concomitant medical conditions, and insufficient hepatic reserve.^5,8^

 Recent studies have shown that the combination of ablative therapies and partial hepatectomy prolongs short- and long-term survival in resectable cases.^3,9^ In unresectable cases, the combination of systemic therapy and ablative therapies prolongs disease-free survival (DFS) and OS with low morbidity rates.^10^ These results have led to increasing interest in ablative therapies and have put forth evidence in support of the widespread and safe use of local ablation in combination with locoregional therapies in a specific subset of patients.^10^ Among these alternative approaches, radiofrequency ablation (RFA) has been used in the past 20 years as a minimally-invasive treatment for local tumor control in patients with CRCLM who have limited hepatic reserve.^11,12^

The present study aimed to determine the long-term outcomes of RFA with respect to OS, DFS, prognostic factors, and complications in patients with CRCLM.

## MATERIAL AND METHODS

Informed consent for RFA was obtained from all patients. The ethics committee of Hacettepe University approved the study for retrospective data analysis (Decision number: GO 13/41). 

### Inclusion and Exclusion Criteria

Patients who were unsuitable for surgical treatment due to comorbidities or lesion location, and were considered to have adequate hepatic tissue following RFA, were enrolled in the study. We used the following inclusion criteria: (i) no evidence of extrahepatic metastasis, (ii) having lesion(s) with a size of ≤6 cm, and (iii) having a metastatic lesion count of ≤6. Considering available evidence suggesting that RFA is ineffective for the treatment of lesions >3-6 cm in size, patients with more than one 6 cm lesion were not treated with RFA. However, if patients had a single lesion with a diameter of 6 cm, they received RFA treatment for all of their lesions (including smaller lesions) in the same session. Since microwave ablation was not available at our center during the study period, all patients had undergone ablation with RFA. The close proximity of the hepatic hilum or main bile duct and the presence of uncorrectable coagulopathy were considered exclusion criteria for RFA. 

### Data Collection

Data collection was performed retrospectively through a review of medical records. Pre-procedural, procedural, and post-procedural data of patients with CRCLM were obtained. Data concerning the use of transarterial chemoembolization (TACE) or transarterial radioembolization (TARE) were not accessible for all patients; thus, these were not recorded.

### Patient Characteristics

Among the 59 patients who had undergone RFA for liver metastases between October 2001 and February 2013, there were 18 (30.5%) women and 41 (69.5%) men. All patients had received adjuvant or neo-adjuvant chemotherapy and/or colon resection prior to RFA treatment; however, none were receiving any of these treatments at the time of RFA application. Patients’ ages ranged from 25 to 87 years, with a mean of 61 years (median, 61 years). Thirty patients had a single lesion, while 29 had multiple lesions. In the first session, 107 lesions were treated. During the follow-up, 20 new lesions developed (10 were intrahepatic new lesions and 10 were local recurrence), and 12 patients were identified to have residual lesions. Therefore, a total of 32 lesions were scheduled for a second session. One patient died before their solitary lesion could receive secondary RFA; thus, data were available for 139 lesions, but a total of 138 lesions were ablated in the first and second sessions. A single patient underwent a third RFA session, but this was not included in the overall analyses. 

Lesion diameters ranged between 1 and 6 cm (mean, 2.45 cm; median, 2 cm) and were categorized with 10-mm increments into 6 groups (Table 1). Lesion sites were not categorized according to any anatomical classification systems (i.e., the Couinaud classification). Twenty-six (44.1%) patients had liver metastasis at the time of initial diagnosis of colon cancer.

### Radiofrequency Ablation Procedure

All procedures were performed under US guidance (Siemens Allegra, Erlangen, Germany), and a 3.5-MHz probe was used. Radiofrequency ablation was performed intraoperatively in 12 (20.3%) patients. According to relevant indications, intraoperative RFA was combined with metastasectomy in 11 patients. In 1 patient, the lesion site was unsuitable for the percutaneous procedure and this patient had also undergone RFA intraoperatively. Percutaneous procedures were performed with the free-hand technique by 3 radiologists experienced in nonvascular interventions at the Nonvascular Interventional Radiology. Radiofrequency ablation procedures were performed using the RITA StarBurst XL and RITA StarBurst Talon (RITA Medical Systems, AngioDynamics, Inc.) thermal ablation probes equipped with a 150 W generator.

### Post-procedural Care and Follow-Up Studies

Following RFA intervention, all patients were monitored at the hospital for at least 1 day and discharged the next day. Contrast-enhanced dynamic liver CT was performed 1 month after the procedure to determine local therapeutic efficacy and technical success. Total ablation was defined with the lack of contrast enhancement around or in the lesion, the presence of smooth and sharp ablation borders, and the measurement of an ablation area larger than the lesion size (on CT images). Patients with residual/recurrent lesions underwent secondary interventions as early as possible. After total ablation, CT and US were performed every 3 months in the first year and every 6 months thereafter. When a suspicious lesion was detected, dynamic liver MRI was performed. The presence of a newly developed focal contrast-enhanced area in the RFA site, the presence of a lesion with new focal contrast enhancement, and the presence of a lesion with increased activity on PET-CT were considered as local recurrence.

### Statistical Analysis

The statistical analyses of the study data were performed using the Statistical Package for Social Sciences for Windows (version 18.0) software package (SPSS Inc.; Chicago, IL, USA). Descriptive statistics included mean or median for continuous variables, and number and percentage (%) for categorical variables. The effects of categorical variables on OS and DFS were determined with Kaplan–Meier survival analysis. A log-rank test was used to determine if there was any significant difference between risk factor categories in terms of mortality. The differences regarding categorical variable frequencies were tested using chi-square tests. *P* <.05 was considered to demonstrate statistical significance.

## RESULTS

A total of 59 patients with 139 liver metastases were evaluated in the study. Since 1 patient died before receiving their second-session RFA, outcome data included 138 lesions treated with RFA in the first and second sessions. Among the 59 patients, 12 (20.3%) underwent RFA simultaneously with the primary surgical intervention (intraoperatively). The indication for intraoperative RFA was a simultaneous application with metastasectomy in 11 patients, while the remaining patients underwent intraoperative RFA because the lesion site was unsuitable for percutaneous application. Apart from this single patient, all lesions could be reached with ultrasonography guidance.

Among the 138 lesions which received RFA, total necrosis was revealed in 127 as demonstrated by CT obtained 1 month after the procedure. The primary success rate for the first session was 94.4% (101 of 107 first-session lesions). Twelve lesions (5.6%) were found to have residue in the first month (CT imaging). The residue rate was 7.5% for lesions with a diameter of ≤3 cm (7 of 93) and 10.8% for those with the size of >3 cm (5 out of 46). Patients with residue were scheduled for a secondary procedure within 1 month. No recurrent residues were detected 1 month after the secondary procedures. The secondary success rate was 98.4% with respect to residual assessment. Among the 12 patients with residue, 1 died due to poor general condition while planning the secondary procedure. One patient showed intrahepatic and extrahepatic new lesions in the first-month CT; thus, no procedure was planned for the residual lesion, and relevant data were not included in the present study.

### Complications, Adverse Events, and Hospital Stay

There was no procedure-related mortality (CIRSE grade 6) in the present study. A total of 4 (6.7%) patients had minor complications (CIRSE grades 1-3). One patient developed minimal pneumothorax, which spontaneously resolved in 2 days (grade 2). Another developed subcapsular hematoma after the procedure, and there was no decrease in hemoglobin level and worsening of bleeding in the control US, and the patient was discharged the next day (grade 1). In 1 of the remaining 2 patients, the control CT showed asymptomatic thrombosis in the portal vein branch of the relevant segment (grade 2). In the last patient, portal vein thrombosis and hepatic vein thrombus developed (grade 3). Following the diagnosis of thrombi, the patient received anticoagulants and was followed as such thereafter. No additional problems occurred (Table 2).

With regard to procedural adverse effects, liver function tests were elevated in 5 (8.4%) patients, mild-to-moderate pain was noted in 9 (15.2%) patients, and fever developed in 3 (5%) patients. All subjects were monitored as in-patients until the symptoms resolved (within 1-4 days in all) (Table 2).

The mean post-procedural hospital stay was 4.4 days, ranging from 1 to 90 days. Patients who underwent intraoperative RFA had a mean duration of hospital stay of 17.4 days, which was significantly longer compared to those with percutaneous RFA (1.2 days) (*P *< .001).

### Survival

The mean follow-up duration after ablation (until death or last follow-up) was 25 months (median, 18.3; range, 6-158 months). Thirty-five patients died during follow-up. The median survival from RFA to death was 34.3 months. The OS rates at 1, 3, and 5 years calculated using the Kaplan–Meier method were 94.9%, 52.5%, and 40.6%, respectively. Median DFS duration was 12 months, with DFS rates at 1, 3, and 5 years being 44.0%, 10.2%, and 6.7%, respectively.

Eleven (18.6%) of the 59 patients were defined to be disease-free at their last visit. Fourteen (23.7%) patients developed new intrahepatic and extrahepatic lesions, while 24 (39.1%) had new intrahepatic lesions only and 11 (18.6%) had new extrahepatic lesions only. Extrahepatic distant metastases involved the lungs and bone. Ten of 24 patients with new intrahepatic lesions developed local recurrence in proximity to the lesion that had received RFA. Fourteen patients had new intrahepatic lesions at non-ablated sites. Twenty-four patients with local recurrence or new intrahepatic lesions at the non-ablated site were treated with RFA twice, and a single patient was treated thrice (the third session not included in outcome analysis).

The median survival was 42 months among patients with a metastasis size of ≤3 cm, whereas this value was 25 months among those with a metastasis size of >3 cm. At 1, 3, and 5 years, patients with metastasis size of ≤3 cm had OS rates of 93.3%, 82.6%, and 59.6%, respectively, whereas patients with a metastasis size of >3 cm had OS rates of 74.8%, 33.2%, and 0% (*P *< .001). Among 27 patients with solitary metastasis, those with metastasis size of ≤3 cm (n = 19) and those with metastasis size of >3 cm (n = 7) had no significant differences with respect to OS (*P *< .430). At 1, 3, and 5 years, patients with solitary metastasis size of ≤3 cm had OS rates of 100.0%, 56.6%, and 35.5%, respectively, whereas OS rates were 80.0%, 55.3%, and 0%, respectively, among patients with solitary metastasis size of >3 cm.

In the Kaplan-Meier analysis, patient age (>60 years vs. <60 years), sex, and metastatic status at the time of diagnosis were not found to be significantly associated with OS. Similarly, the presence of new intrahepatic lesions or local recurrence at follow-up was not significantly associated with OS.

Patients with solitary metastasis had significantly longer OS compared to those with multiple metastatic lesions (*P *< .008). Moreover, patients with extrahepatic involvement during follow-up had significantly lower OS rates compared to those without extrahepatic involvement (*P *< .005).

Patient age (>60 years vs. <60 years), sex, and metastatic status at the time of diagnosis were not significantly associated with DFS values. However, patients with solitary metastasis had significantly longer DFS compared to those with multiple metastatic lesions at initial RFA treatment (*P *< 0.001).

## DISCUSSION

Our study revealed OS rates similar to the majority of the literature. In 59 patients and 138 lesions treated with RFA, the OS rates at 1, 3, and 5 years were 94.9%, 52.5%, and 40.6%, respectively. Solbiati et al^13^ treated 117 patients with CRC who had 179 liver metastases with diameters ranging from 0.6-9.6 cm, the authors revealed survival rates of 93.2%, 69%, and 35% at 1, 3, and 5 years, respectively. In our study, the median DFS was 12 months, with rates at 1, 3, and 5 years being 44.0%, 10.2%, and 6.7%, respectively. In 2012, Solbiati et al^14^ reported the findings of a 202-lesion study comprising a 10-year follow-up, making it the RFA study with the longest follow-up period. They reported 1, 3, and 5-year survival rates of 98%, 69%, and 47%, respectively. The 5-year survival rates shown by this study support our findings. Recent studies have indicated that survival rates were boosted from approximately 22% to 44% compared to former studies; this difference was attributed to the development of effective chemotherapeutics, widespread use of TACE treatment, and introduction of radioembolization into available treatment options for diffuse metastatic disease.^14^ Recently, Hof et al^15^ reported the outcomes of surgery and/or RFA and 5-year OS rates of 51.9% for surgery and 53% for RFA.

The randomized controlled trial, called the CLOCC study, compared chemotherapy alone and a combination of RFA and chemotherapy in patients with unresectable metastases and reported that combination therapy prolonged progression-free survival from 9.9 months to 16.8 months and OS from 57.6% to 61.7% in a period of 30 months. They revealed that local ablative therapies, including RFA, were superior to systemic chemotherapy alone and increased OS in the treatment of CRCLM in long-term follow-up, as demonstrated by OS rates of 56.9%, 43.1%, and 35.9% at 3, 5, and 8 years, respectively, especially among patients followed with aggressive local therapies.^10^

Compared to surgery, thermal ablation is associated with a lower complication rate and better health-related quality of life, while its cost is also lower with a shorter duration of hospital stay. Studies have shown that ablation can be preferred over surgery in patients with unresectable (due to size or localization) metastases or resectable metastases with a higher surgical risk. He et al^16^ reported no OS difference between hepatic resection and ablation at 1, 3, and 5 years, but recurrence-free survival rates in the resection group were 90.6%, 56.3%, and 28.1%, respectively, and those in the RFA group were 76.1%, 23.8%, and 4.8%, respectively, with the differences being significant between the 2 groups. In a study reported by Hof et al^15^ similar survival rates were reported in both groups, whereas the resection group was superior in terms of DFS. This same study found that the OS rates of the resection group were 87.5%, 53.1%, and 31.3%, respectively, while our study found corresponding rates of 94.9%, 52.5%, and 40.6%, respectively.^15^ Our study also found similar OS when compared to resection. Some researchers tend to prefer RFA despite higher recurrence rates (but similar OS) due to various reasons, including higher long-term success regarding pain alleviation, lower costs, and shorter hospital stays. However, when local recurrence develops, repeat interventions with RFA can be carried out easily, apparently without compromising the long-term outcome. Nevertheless, a meta-analysis in 2019 showed that resection, when possible, is superior to RFA for OS and DFS.^17^

Although there is no clearly defined upper limit to the number of lesions that can be treated with RFA, it has been shown that local recurrence and survival rates are correlated with the number of treated tumors. Within this group, the best outcomes are achieved with the treatment of solitary lesions. While the outcomes remain relatively favorable for lesion counts of 1-3, the poorest outcomes are observed when more than three lesions are treated. A meta-analysis of studies on solitary metastases (<3 cm in size) showed that hepatic resection is superior to RFA in terms of both local recurrence and OS.^17^ The most recent meta-analyses on this topic report similar short- and mid-term survival, but worse long-term survival among RFA recipients compared to those who underwent resection.^18^ In a prospective study by Nan et al, it was shown that the median DFS was 20 months for metastases smaller than 3 cm that was treated with RFA, while it was 37 months in those who underwent hepatectomy. The 1-, 3- and 5-year recurrence-free survival rates for the resection group were 90.6%, 56.3%, and 28.1%, whereas those of the RFA group were 76.1%, 23.8%, and 4.8%, respectively; significant differences in these values were noted between the groups.^16^ Some retrospective studies have shown comparable survival between resection and ablation, particularly when ablation is applied to smaller tumors <3 cm,^19-21^ and RFA only versus RFA + resection have been shown to demonstrate similar lengths of survival.^22^ Very recent studies by Třeška et al (RFA vs. resection)^23^ and Yang et al (rectal metastasis vs. colorectal metastasis)^24^ have also revealed similar lengths of short- and mid-term survival with RFA in their 10-year and 3-year studies. When RFA is applied to a tumor with a diameter of <3 cm with curative intent, a study by Shin and colleagues showed comparable results regarding DFS and OS between hepatic resection and RFA.^19^ Additionally, a recent study by Marchese et al^25^ evaluated ablative treatment (microwave or RFA) applied to patients with smaller (<40 mm) solitary lesions and compared outcomes to those that had undergone resection. Survival results pertaining to ablative treatments were largely similar to resection outcomes and showed 1-, 3-, and 5-year OS rates of 97.4%, 84.9%, and 74.9%, respectively, while DFS rates for the same periods were 77.9%, 47%, and 38.9%, respectively.^25^ Although only 13 patients had undergone RFA and 16 had undergone microwave treatment, these results support the notion that ablative treatments are especially effective on smaller and/or solitary metastatic lesions. In a very recent randomized controlled trial, RFA and microwave ablation were found to yield similar positive outcomes in the treatment of relatively small CRCLMs.^26^ These results are mirrored by other recent randomized controlled trials which included patients with hepatocellular cancer or liver metastasis (1.5-4 cm in diameter), showing similar capabilities of microwave and RFA.^27^ In line with previous observations, our study found better survival among patients with a lesion size of <3 cm compared to those with a lesion size of >3 cm. However, OS rates of metastases that were <3 cm and solitary were not significantly different than that of solitary metastases with a size of >3 cm (n = 7), which can be attributed to the limited number of cases.

In the present series, 20 lesions with an intrahepatic new lesion or local recurrence were treated with 2 RFA sessions and 1 was treated with 3 RFA sessions. Radiofrequency ablation is a repeatable procedure for patients with recurrence as long as liver function permits. This shows that repeated RFA procedures are not as invasive as to cause liver injury. We noted that our primary technical success rate was 94.4% in the first month, as indicated by control abdominal CTs. The secondary technical success rate after the second session of ablation was 98.4%. According to a guideline published by Crocetti et al^28^ in 2010, which included criteria for technical improvement of RFA use for hepatic tumors, a rate of ≥90% ablation was deemed to be acceptable to define technical success.

Our study’s findings show that RFA is effective and safe for CRCLM treatment. Four (6.7%) of treated patients developed minor complications (CIRSE grades 1 and 2). No procedural death occurred (CIRSE grade 6). In an early study, Livraghi et al^29^ reported a mortality rate of 0.3% in 2320 patients and cited RFA as a safe method. They also reported that early major complication rates of RFA ranged from 2.2% to 3.1% and revealed that these consisted of intraperitoneal bleeding requiring transfusion, hepatic abscess, hemothorax, and intestinal perforation. Among the patients in our study, 1 subject had a subcapsular hematoma and mild intraperitoneal hematoma but did not require transfusion. Apart from this, a case of pneumothorax was conservatively managed as it involved <25% of the lung. Portal vein thrombus and hepatic vein thrombus that developed in the two remaining patients did not cause mortality or morbidity, despite the fact that the literature shows that portal vein thrombus is a cause of death after RFA. All 4 complications were deemed minor complications as they resolved spontaneously and required no additional intervention. The rate of minor complications in the literature ranges from 1% to 8.7%.^24,30^

Our study had some limitations. First, our sample size was small. Second, the medical approach and chemotherapy regimens have changed considerably over the years, which may have been influential on survival analyses considering that the CRCLM patients in this study had been diagnosed between 2001 and 2013. This is particularly pertinent when data from meta-analyses show that patients who received RFA in later years (2011-2018) had significantly better survival compared to those who received RFA in earlier years (2003-2010).^12^ Third, our study was conducted with a retrospective design and is subject to all associated limitations. However, we believe that our study can be a good contribution to the literature which is largely comprised of studies with heterogeneous patient groups.

In conclusion, this retrospective study demonstrates that lesion diameter (>3 cm), multiple lesions, and the development of extrahepatic recurrence are the strongest risk factors associated with poor long-term outcomes and survival in patients with CRCLM. Currently, RFA is used as a safe and effective method with 5-year survival values comparable to surgical resection. Radiofrequency ablation remains a reliable treatment for patients with unresectable CRCLM since it prolongs OS and DFS.

## Figures and Tables

**Table 1.** Lesion Characteristics

Lesion Size (mm)	Lesion Count
0-11	34
11-20	26
21-30	33
31-40	40
41-50	3
51-60	3

**Table 2.** Complications Classified According to CIRSE Quality Assurance Document and Standards for Classification of Complications

Complications	n	CIRSE Classification	Comments
Hemorrhage	1	1	Self-limiting, no treatment
Pneumothorax	1	1	No treatment; observation
Portal vein thrombosis (asymptomatic)	1	2	No treatment; observation
Portal vein thrombosis	1	3	Anticoagulant treatment
